# Indocyanine green matching phantom for fluorescence-guided surgery imaging system characterization and performance assessment

**DOI:** 10.1117/1.JBO.25.5.056003

**Published:** 2020-05-21

**Authors:** Alberto J. Ruiz, Mindy Wu, Ethan P. M. LaRochelle, Dimitris Gorpas, Vasilis Ntziachristos, T. Joshua Pfefer, Brian W. Pogue

**Affiliations:** a Dartmouth College, Thayer School of Engineering, Hanover, New Hampshire, United States; b Institute of Biological and Medical Imaging, Helmholtz Zentrum München, Munich, Germany; c Technical University Munich, Helmholtz Zentrum Munich, Munich, Germany; d U.S. Food and Drug Administration, Center for Devices and Radiological Health, Rockville, Maryland, United States; e Geisel School of Medicine, Department of Surgery, Hanover, New Hampshire, United States

**Keywords:** fluorescence-guided surgery, tissue simulating phantoms, imaging standard, surgery, indocyanine green

## Abstract

**Significance:** Expanded use of fluorescence-guided surgery with devices approved for use with indocyanine green (ICG) has led to a range of commercial systems available. There is a compelling need to be able to independently characterize system performance and allow for cross-system comparisons.

**Aim:** The goal of this work is to expand on previous proposed fluorescence imaging standard designs to develop a long-term stable phantom that spectrally matches ICG characteristics and utilizes 3D printing technology for incorporating tissue-equivalent materials.

**Approach:** A batch of test targets was created to assess ICG concentration sensitivity in the 0.3- to 1000-nM range, tissue-equivalent depth sensitivity down to 6 mm, and spatial resolution with a USAF test chart. Comparisons were completed with a range of systems that have significantly different imaging capabilities and applications, including the Li-Cor^®^ Odyssey, Li-Cor^®^ Pearl, PerkinElmer^®^ Solaris, and Stryker^®^ Spy Elite.

**Results:** Imaging of the ICG-matching phantoms with all four commercially available systems showed the ability to benchmark system performance and allow for cross-system comparisons. The fluorescence tests were able to assess differences in the detectable concentrations of ICG with sensitivity differences >10× for preclinical and clinical systems. Furthermore, the tests successfully assessed system differences in the depth-signal decay rate, as well as resolution performance and image artifacts. The manufacturing variations, photostability, and mechanical design of the tests showed promise in providing long-term stable standards for fluorescence imaging.

**Conclusions:** The presented ICG-matching phantom provides a major step toward standardizing performance characterization and cross-system comparisons for devices approved for use with ICG. The developed hybrid manufacturing platform can incorporate long-term stable fluorescing agents with 3D printed tissue-equivalent material. Further, long-term testing of the phantom and refinements to the manufacturing process are necessary for future implementation as a widely adopted fluorescence imaging standard.

## 1 Introduction

Fluorescence imaging in surgery allows for visualization of biologically relevant markers that are indicated to guide rapid decision-making during the procedure.^1^^–^^3^ The use of fluorescence-guided surgery (FGS) has been growing at a steady pace over the past 70 years, with rapid expansion over the last decade as the imaging technologies have matured to enable seamless integration into the clinical workflow.^2^ Indocyanine green (ICG) is by far the most widely used tissue perfusion agent and cardiac flow indicator within FGS.^4^ The success of ICG within fluorescence imaging can be attributed to its absorption and emission in the near-infrared (NIR) range, its low toxicity, and its history of use within medicine for over half a century.^2^^,^^4^^–^^6^ As the field of FGS expands, there is a wider range of imaging systems that are indicated for the same uses, such that there is a compelling need for standards that enable system characterization, performance monitoring, and intersystem comparisons or calibration.^7^^–^^10^ The performance of imaging systems can affect surgical decision making,^7^ so knowledge of their performance capabilities is especially relevant given the real-time decisions that are made with these systems during surgical procedures.

Over the past two decades, research on fluorescent imaging phantoms has undergone significant advances for applications in testing system designs, optimizing signal to noise in existing systems, routine quality control, and cross-system comparisons.^7^^,^^10^ To create stable imaging phantoms, it is important to address both mechanical and photostability issues. Possible substrates and fluorophores that have been studied for long-term stability include epoxy,^11^ polyurathane,^12^^,^^13^ quantum dots,^14^^,^^15^ and polymers.^16^ In recent years, 3D printing has also been used for tuning optical parameters and mimicking anatomical structures.^17^^–^^20^ The main roadblocks associated with developing widely adopted fluorescent standards involve the manufacturing of application-specific spectral characteristics alongside the need for long-term photostability, manufacturing scalability, and reproducibility.

Here a long-term stable fluorescence imaging standard is advanced that incorporates an ICG-equivalent dye with matching spectral behavior, which can be used to test aspects of commercially available imaging systems indicated for use with ICG. The design used was based upon the imaging tests outlined by Anastasopoulou et al.^21^ and Gorpas et al.^8^^,^^22^ in combination with the 3D printing approach presented by Liu et. al.^18^ The imaging standard is composed of three tests: (1) varying concentration sensitivity (0.3 to 1000 nM), (2) tissue-equivalent-depth sensitivity (0.2 to 6 mm), and (3) fluorescence spatial resolution (using a negative 1951 USFA test chart). These test targets allow for ICG-specific system characterization, performance monitoring, and cross-system sensitivity comparisons. The developed fluorescence standard manufacturing platform can incorporate 3D printing with tissue-equivalent layers along with long-term stable fluorophores, with potential applications beyond ICG imaging.

## 2 Materials and Methods

### 2.1 Indocyanine Green Matching Standard

**Fig. 1:**
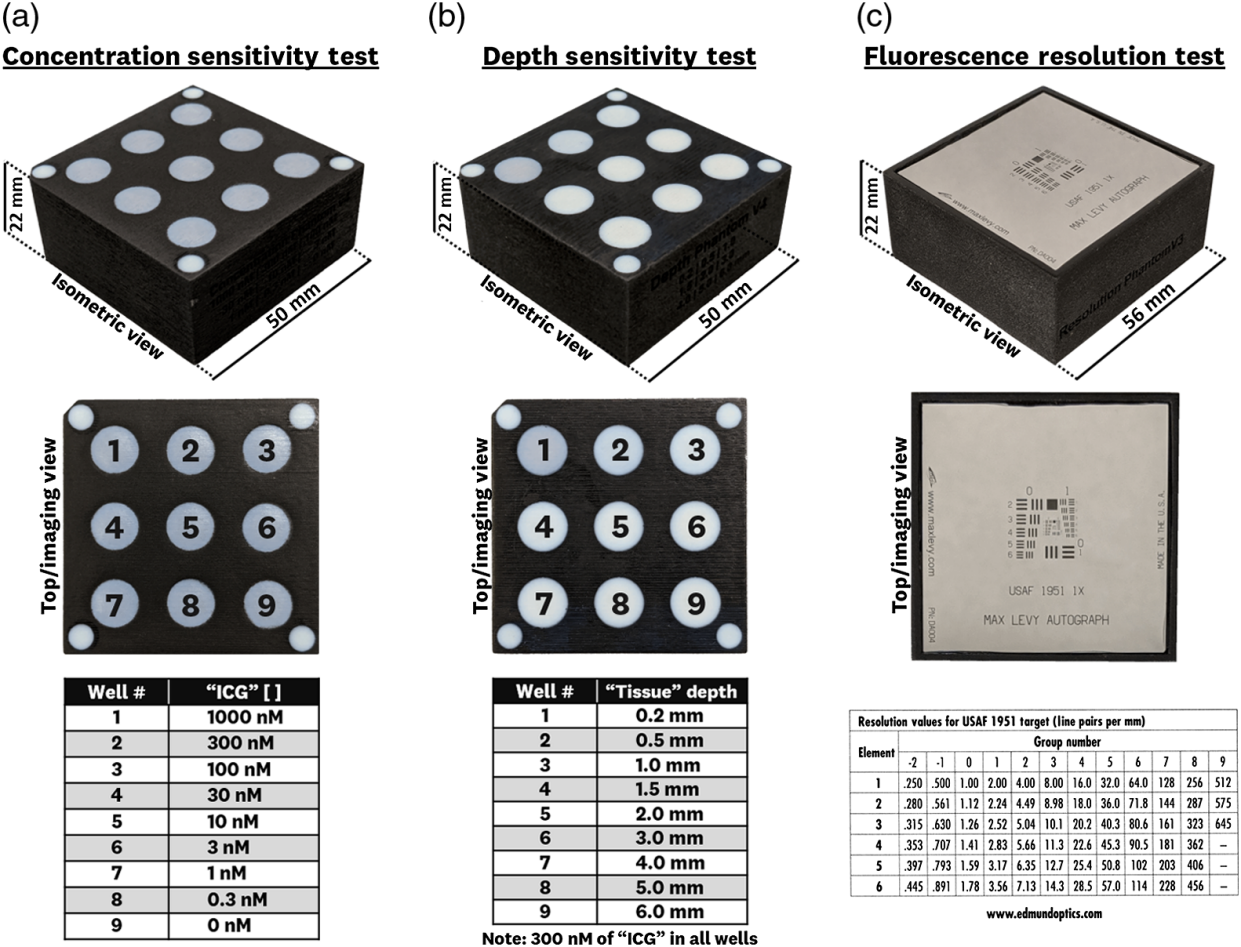
ICG-equivalent fluorescence test standards, composed of a mechanical substrate and a fluorophore–polyurethane mixture that uses IR-125 laser dye as the ICG-equivalent, hemin as an absorbing agent, and ${\mathrm{TiO}}_{2}$ as a scattering agent: (a) concentration sensitivity test that uses varying fluorophore concentrations (0.3 to 1000 nM) for testing a system’s ICG fluorescence sensitivity performance, (b) depth sensitivity test with varying tissue-equivalent depths (0.2 to 6 mm, using ICG-equivalence at 300 nM) used for characterizing a system’s depth-signal curve, and (c) fluorescence resolution test using a negative USAF 1951 target with ICG equivalence at 300 nM to determine the fluorescence imaging resolution.

The three tests composing the ICG-matching fluorescence imaging standard are shown in Fig. 1. The concentration sensitivity test [Fig. 1(a)] uses nine wells of varying concentrations (0.3 to 1000 nM) alongside a control well with no fluorescing agent. The depth sensitivity test [Fig. 1(b)] uses nine wells of varying tissue-equivalent depths (0.2 to 6 mm) with a constant 300-nM ICG-equivalent concentration. The fluorescence resolution test [Fig. 1(c)] uses a USAF 1951 negative resolution target with a homogeneous background of 300-nM ICG-equivalent concentration. These three tests are designed to assess the full dynamic range of imaging systems used for imaging ICG for *in vivo* and *ex vivo* applications in both preclinical and clinical settings.

**Fig. 2:**
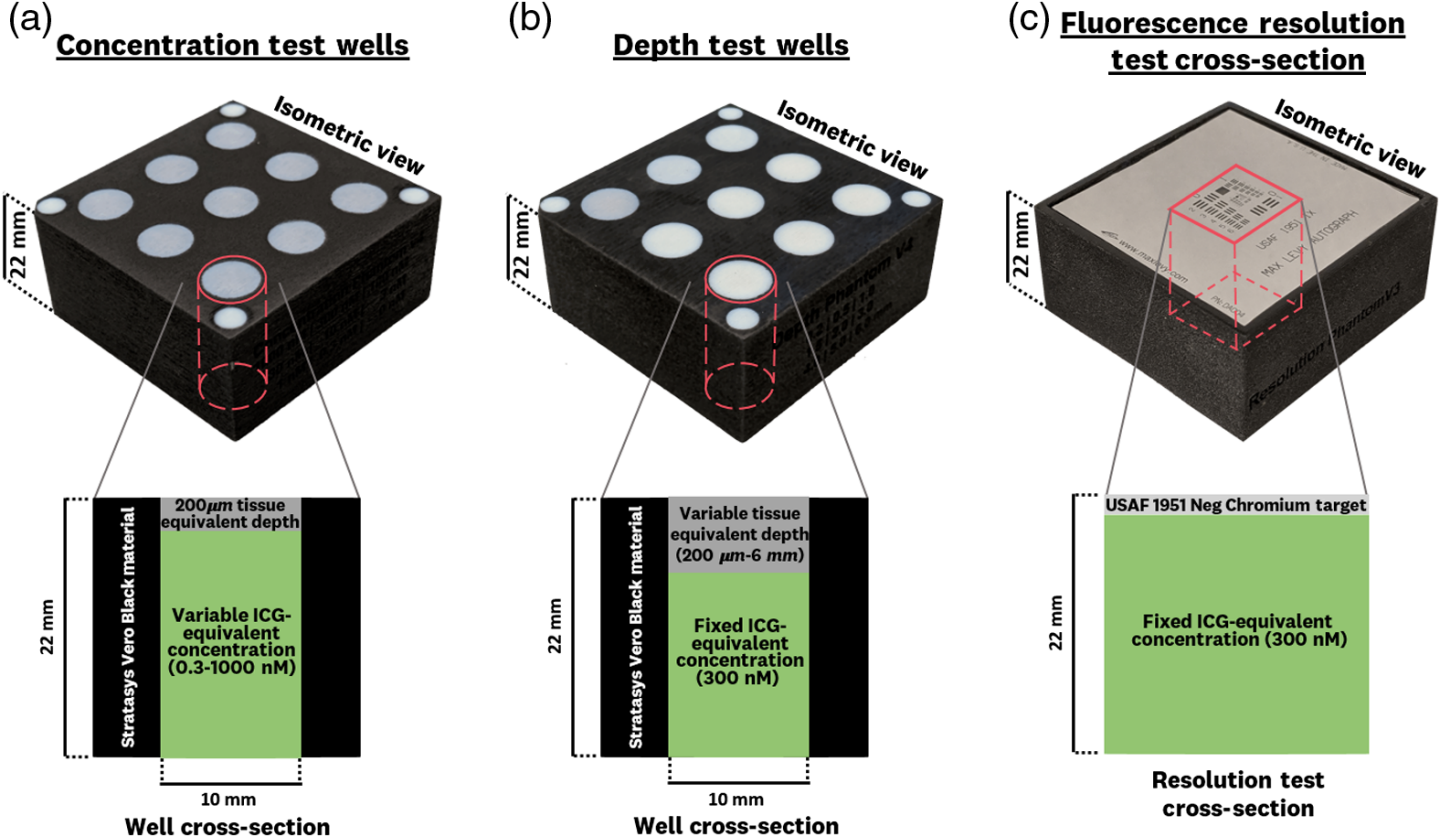
Vertical cross sections of each test composing the ICG-matching standard: (a) concentration test, (b) depth test, and (c) fluorescence resolution test. The specific material compositions of the molds and ICG-equivalent material are described in Secs. 2.1.1 and 2.1.2.

Each test is composed of a 3D printed mechanical mold and the ICG-matching fluorescent mixture. The details of these two components are described in Secs. 2.1.1 and 2.1.2. Cross sections of each test are shown in Fig. 2, where the 3D printed materials, ICG-matching material, and USAF 1951 target are layered to test the concentration sensitivity, depth-signal curve, and resolution of each system. The four smaller wells found on the corners of the concentration and depth sensitivity tests are composed of high-scattering, nonfluorescent material to measure excitation backscatter noise.

#### 2.1.1 3D printed molds

Each of the three fluorescence tests use a 3D printed mold to achieve long-term mechanical stability and allow for the combinations of materials with varying optical properties. The 3D printed molds are polyjet printed by Stratasys Direct Inc. (Los Angeles, California) using Vero White, Vero Black, and RGDA8510 materials to generate mechanically stable molds that simultaneously incorporate tissue equivalent and optically opaque material to mimic tissue optical properties and prevent optical cross talk from adjacent wells. The Stratasys RGDA8510 “digital” material, which combines Vero White and Tango Black material at a ∼95/5% ratio, is used to mimic tissue-equivalent optical properties with measured absorption ($\mu_{a}$) and reduced scattering ($\mu_{s}^{\prime }$) coefficients of 0.361 and $5.355\;{\mathrm{cm}}^{-1}$, respectively. The absorption and reduced scattering coefficients were measured at 731 nm using a Refect RS spatial frequency domain imager (Modulim, Irvine, California) on a $50\times 50\times 22\;{\mathrm{mm}}^{3}$ block of the RGDA8510 material. All three tests use Vero Black as the over-mold material to prevent optical cross talk during measurement. The four smaller wells found on the corners of the concentration and depth sensitivity tests are composed of Vero White material to enable measurement of the excitation backscatter noise.

The concentration sensitivity test [Fig. 2(a)] uses Vero Black as the mold material and incorporates a 200-μm tissue-equivalent layer (RGDA8510) at the top of each well to allow for the deposition of the fluorescent polyurethane mixture on the underside when the mold is flipped. The depth sensitivity test [Fig. 2(b)] uses Vero Black as the mold material and incorporates varying thicknesses of the tissue-equivalent material (RGDA8510) at the top of each well. The fluorescence resolution test [Fig. 2(c)] uses Vero Black as the mold material and is designed to allow for the fixed placement of the negative 1951 USAF resolution target (DDA004, Max Levy Autograph, Inc., Philadelphia, Pennsylvania) and deposition of the homogeneous fluorescent mixture [Fig. 2(c)]. It is worth noting that the 200-μm tissue-equivalent layer thickness was chosen as the smallest thickness that allowed for the deposition of the fluorescent mixture while providing manufacturing reproducibility.

#### 2.1.2 ICG-equivalent fluorescent mixture

To simulate ICG spectral properties in tissue, a fluorescent mixture is manufactured with IR-125 laser dye (Exciton Inc., Lockburne, Ohio), a two-part polyurethane mixture (WC-783 A/B, BJB Enterprises, Tustin, California), hemin (H9039, Sigma Aldrich, St. Louis, Missouri), and ${\mathrm{TiO}}_{2}$ (248576, Sigma Aldrich, St. Louis, Missouri). Hemin is used as the absorbing agent and ${\mathrm{TiO}}_{2}$ as the scattering agent. The concentrations of hemin and ${\mathrm{TiO}}_{2}$ used are 20  μg/g and 0.66  μg/g, respectively, by which, as per Anastasopoulou et al.,^21^ they simulate an absorption coefficient of $0.25\;{\mathrm{cm}}^{-1}$ and reduced scattering coefficient of $6.6\;{\mathrm{cm}}^{-1}$ at 750 nm. Hemin, an iron-containing porphyrin, is used as the absorbing agent since it simulates the absorption of blood.^7^ The wells within each test are filled with the fluorescent mixture described above in accordance with ICG-equivalent concentrations listed in Fig. 1, resulting in the well cross sections shown in Fig. 2. The two-part polyurethane mixture is used as a solution that can be poured into the wells of the 3D printed molds and result in a rigid substrate that can provide long-term mechanical and optical stability.^12^ This two-part polyurethane is mixed at a ratio (A/B) of 100/90 by weight and 100/94 by volume, where part A and part B are the isocyanate and polyol mixtures, respectively.^23^^,^^24^ The density of the cured polyurethane mixture is 1.05  g/mL, and the molar mass of IR-125 is 774.97.

**Fig. 3:**
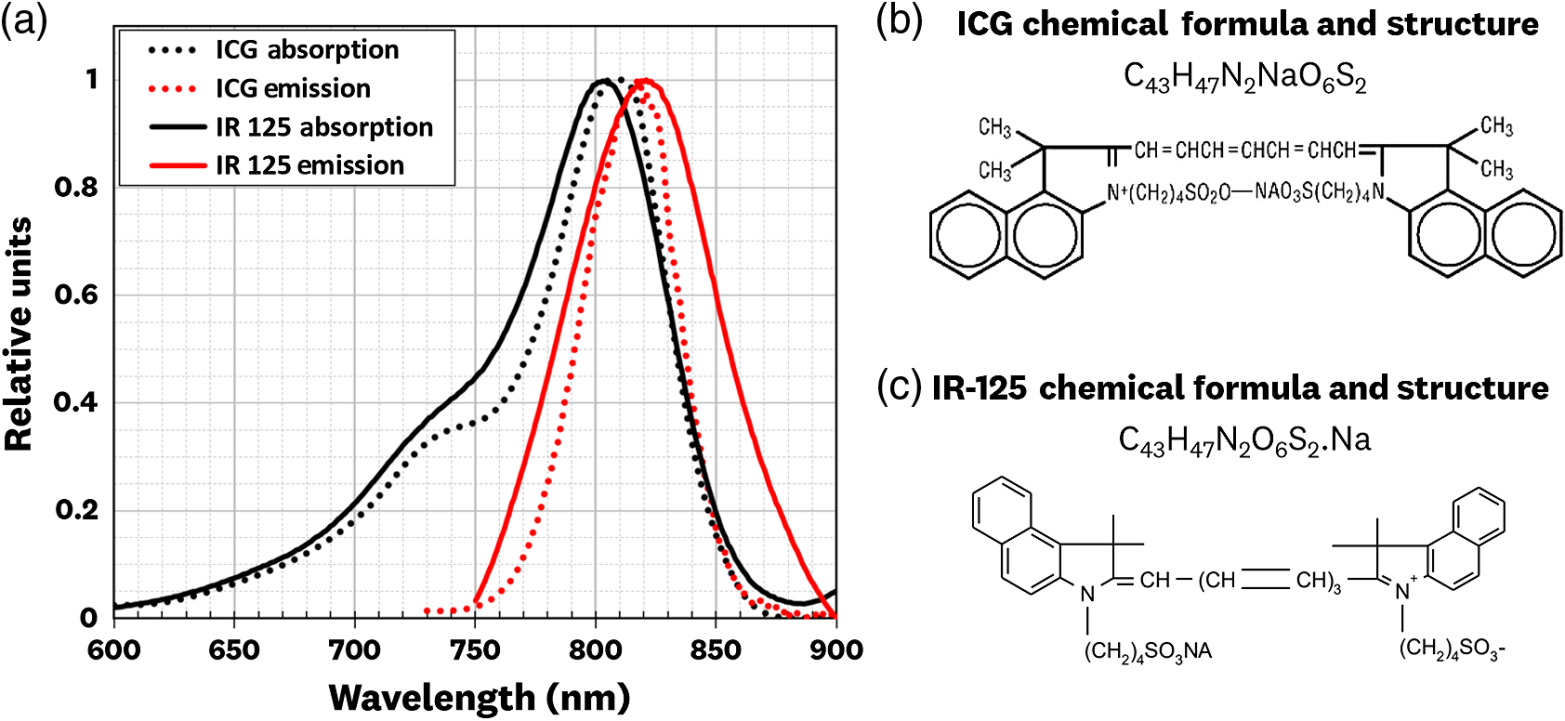
(a) Absorption and emission spectra for ICG (in plasma)^25^^,^^26^ and IR-125 laser dye (in polyurethane), showing similar spectral behavior. The overlap in the absorption and excitation between the two fluorophores showcases IR-125’s ability to serve as an ICG-equivalent fluorophore. (b) Chemical formula and structure of ICG as listed in an FDA’s new drug application.^27^ (c) IR-125 chemical formula and structure as per manufacturer specification.^28^

Figure 3(a) shows the absorption and emission spectra of IR-125 suspended in the cured polyurethane alongside published spectra of ICG in plasma.^25^^,^^26^ The IR-125 spectra were collected at a 3000-nM concentration using a spectrofluorometer (FluoroMax-4, Horiba Ltd., Japan); a single scan was performed at 1-nm steps with a high signal-to-noise ratio. The IR-125 spectra show good overlap with ICG-in-plasma spectra, showcasing IR-125’s ability to serve as an ICG-equivalent fluorophore. The similarities in absorption and emission between IR-125 and ICG come from both being cyanine dyes with similar molecular structures [Figs. 2(b) and 2(c)], resulting in similar quantum yields of 0.13 in dimethyl sulfoxide (DMSO).^29^ From a historical perspective, it is worth noting that ICG was first developed at the Mayo Clinic in the late 1950s for medical applications,^30^^–^^32^ whereas IR-125 was first developed as a laser dye in 1974,^33^ with the Eastman Kodak Company (Rochester, New York) taking part in the development of both dyes.

IR-125 photostability has been previously investigated and shows excellent resistance to bleaching.^34^ Furthermore, photostability of polyurethane has also been studied for periods exceeding 1 year,^12^ indicating that it can be used to provide long-term mechanical and optical stability in phantoms. Preliminary measurements indicate that the ICG-matching standards presented here have not undergone mechanical changes and have only minor decay in optical characteristics.

The measured fluorescence stability plots can be found in Fig. S1 in the Supplementary Material. Optical stability of the fluorescent mixture was assessed on a daily basis over a 2-month period using the Stryker^®^ Spy Elite imaging system and showed a linear decay of 0.09%/week. Extrapolating this data would suggest a decay of <5% for the signal over the year. Optical stability of the finalized phantom design was measured using a Li-Cor^®^ Odessey CLx imaging system, where fluorescence intensity showed variations that did not exceed 5% over a 5-month period; these phantoms were stored in a black box container at room temperature, where they are exposed to light only during the fluorescence measurement. Further characterization of the fluorescent decay and long-term stability is ongoing to enable the ability to calibrate intensities of the phantom over time.

### 2.2 Manufacturing

**Fig. 4:**
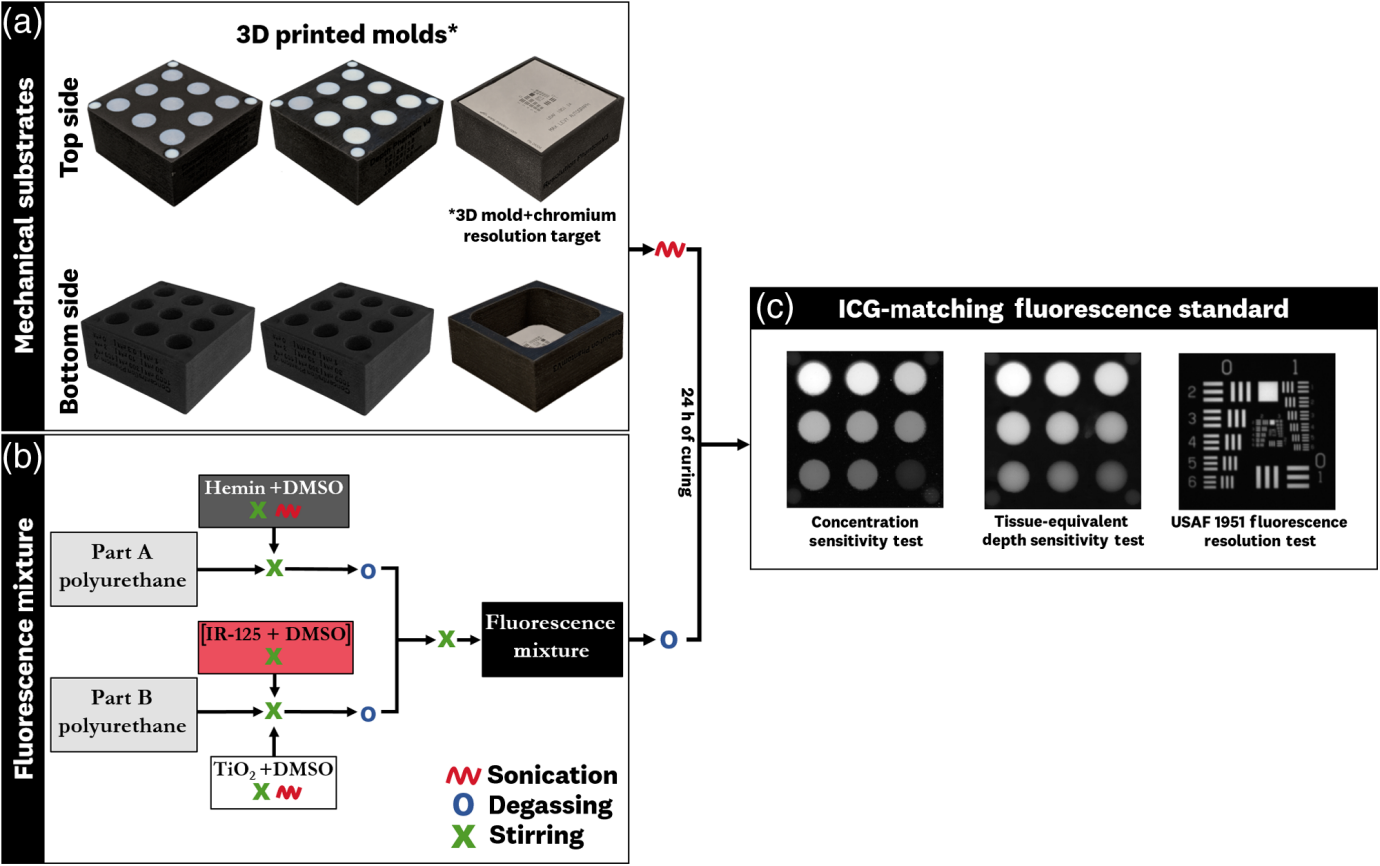
Manufacturing overview for the ICG-equivalent fluorescence test standards: (a) 3D printed molds are polyjet printed by Stratasys Manufacturing Inc. using Vero White, Vero Black, and RGDA8510 materials to generate mechanically stable molds that incorporate tissue-equivalent material and prevent optical cross talk from adjacent wells; these molds are sonicated for 40 min and left to dry over 24 h before depositing the fluorescence mixture. (b) Fluorescence mixture manufacturing procedure involving the use of a two-part polyurethane, IR-125, hemin, and ${\mathrm{TiO}}_{2}$. The fluorescent mixture is deposited into the 3D printed molds and left to cure for 24 h, resulting in (c) the complete ICG-equivalent fluorescence standard (as imaged on the Li-cor^®^ Odyssey Clx using the 800 channel).

A visual overview of the manufacturing process for the ICG-matching standard is provided in Fig. 4. The 3D printed molds [Fig. 4(a)] are prepared for the deposition of the fluorescent polyurethane mixture by sonicating in water for 40 min and left to dry under LED room light for 24 h; these preparation steps ensure that any uncured 3D printed material is removed from the molds, preventing the creation of further air bubbles during polyurethane curing. Manufacturing of the ICG-matching fluorescent mixture [Fig. 4(b)] begins with the creation of stock solutions of hemin, ${\mathrm{TiO}}_{2}$, and IR-125 in DMSO. The concentrations used are 1  mg hemin/262.5  μL DMSO, $1\;\mathrm{mg}\;{\mathrm{TiO}}_{2}/6\;\mu L$ DMSO, and nine separate, serially diluted, IR-125 solutions of respective concentrations to deposit 10  μL of solution in 3 mL of polyurethane. These ratios are chosen as a balance between optimal solubility and minimization of added DMSO volume to reduce alterations to the curing process. These solutions are incorporated into part A and part B of the polyurethane and degassed to remove any trapped air. Part A and B are combined, mixed, degassed, and left to cure for 40 min before deposition. Each fluorescent mixture is then deposited into the wells of the 3D printed molds and left to cure over 24 h, resulting in the ICG-matching fluorescence standard tests [Fig. 4(c)].

### 2.3 Assessing Phantom Performance

**Fig. 5:**
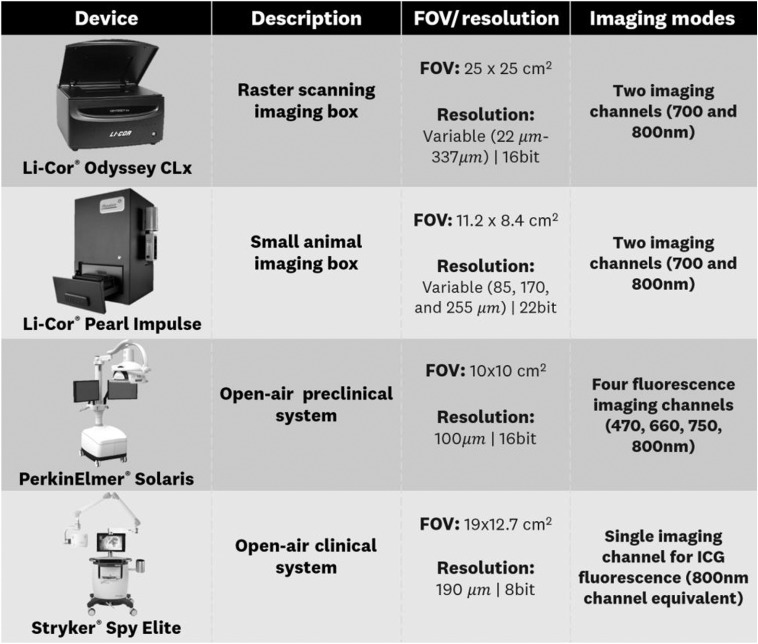
Imaging systems summary used for evaluating ICG-matching standard performance.

To test the performance of the ICG-matching standard in assessing the full dynamic range sensitivity of commercially available systems, four representative devices were chosen. These devices allow for comparison of closed box versus open air systems, wide-field imaging versus raster scanning, and preclinical versus clinical devices. Figure 5 summarizes the characteristics of each system including resolution and imaging modes. The listed resolution for the PerkinElmer^®^ Solaris and Stryker^®^ Spy Elite is calculated from field-of-view and pixel count specifications provided by the manufacturers.

The three preclinical systems (Li-cor^®^ Odyssey CLx, Li-cor^®^ Pearl Impulse, and PerkinElmer^®^ Solaris) can image ICG through their “800 channel” imaging mode, whereas the clinical system (Stryker^®^ Spy Elite) has a single imaging channel specific to ICG fluorescence. Each of these devices automatically generates image acquisitions without the ability of the user to change parameters such as exposure time. This means that the results presented below are representative of the “off-the-shelf” performance for each system as per manufacturer specifications.

The average pixel value for each well is calculated by measuring a centered region of interest (ROI) of ∼8  mm diameter to minimize any boundary-absorption effects from the optically opaque mold. The linearity range associated with each device is calculated based on measured signals that are above the noise floor and unsaturated. Noise floor values can be quantitatively assessed in the log10–log10 and log10–linear plots for the concentration and depth tests, respectively. Saturated values are determined by comparing the measured pixel output to the maximum bit output of each system. A formalized mathematical algorithm should be developed in the future to ensure true cross-system comparison for calculated noise-floor and saturated values. The measured resolution is calculated by determining the line pairs with the lowest spatial frequency that satisfy the Rayleigh criterion. A single ICG-matching standard set is used for the assessment of system performance. Furthermore, we explore manufacturing reproducibility among five sets of fluorescent standards by imaging the tests on the Li-cor^®^ Odyssey Clx given its ability to measure signal above noise floor for all of the fluorescent wells.

## 3 Results

Each of the three tests composing the ICG-matching standard was tested on the four systems listed in Fig. 5. The results for each test are presented in the following sections.

### 3.1 Concentration Sensitivity Testing Standard

**Fig. 6:**
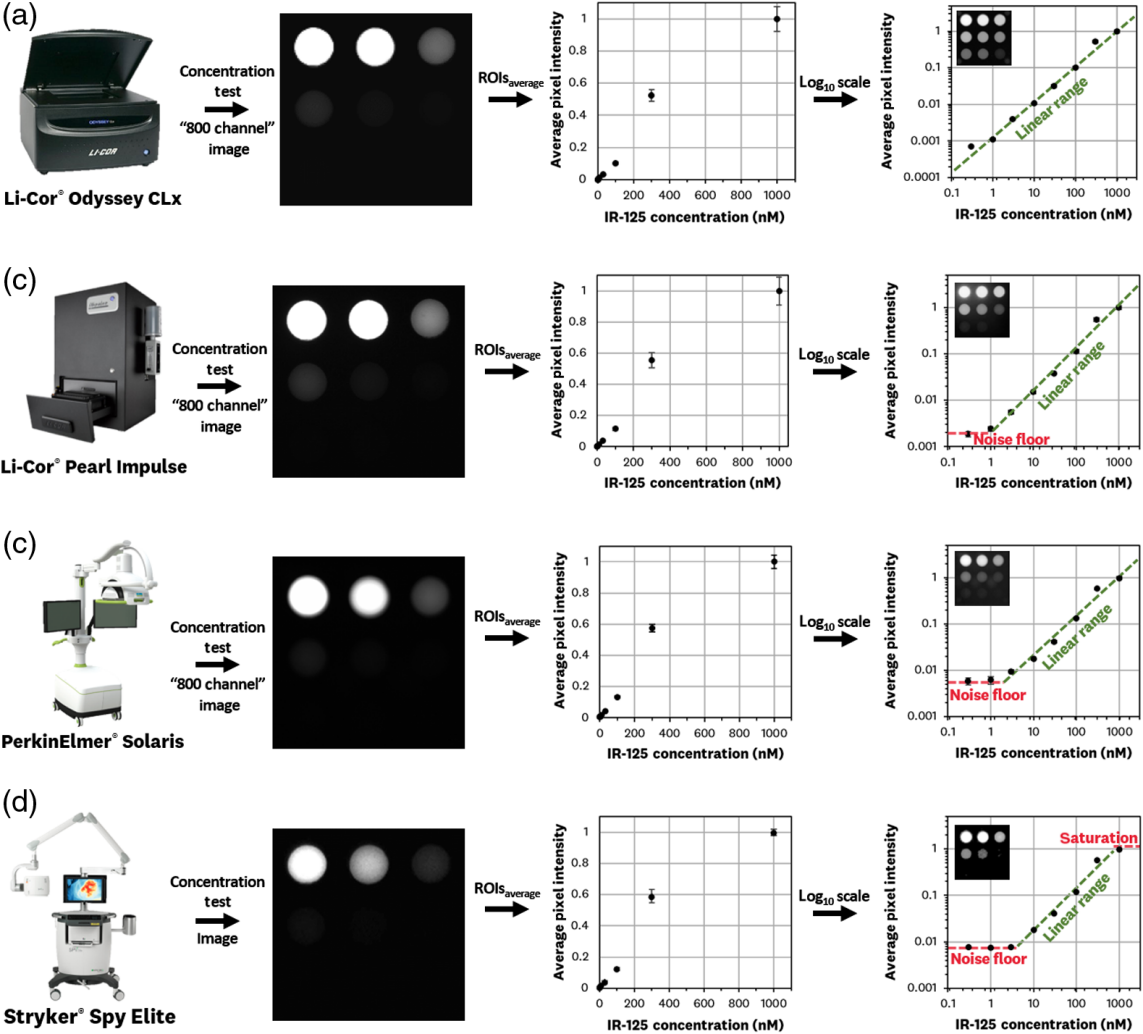
System imaging results for the concentration sensitivity test standard. The test is imaged for the ICG-specific channel of each system and average pixel values of the ROIs corresponding to each well are calculated, normalized, and plotted; the log10–log10 scaling plot of these average pixel values allows for the observation of a system’s noise floor, usable linear range, and saturation over the range of 0.3 to 1000 nM. Images, calculated ROI values, and linearity range for (a) Li-Cor^®^ Odyssey CLx “800 channel,” (b) Li-Cor^®^ Pearl Impulse “800 channel,” (c) PerkinElmer^®^ Solaris “800 channel” image, and (d) Stryker^®^ Spy Elite. The images on the top left corner of the log10–log10 graphs are the logarithmic pixel representation of the respective fluorescence image.

The resulting images and ROI average pixel calculations for the concentration sensitivity test are shown in Fig. 6. The average pixel values are normalized to the 1000-nM concentration well for each system. Plotting the average values in a log10–log10 plots allows for a visual assessment of a system’s noise floor, usable linear range, and saturation over the 0.3- to 1000-nM range. The Li-Cor^®^ Odyssey Clx [Fig. 6(a)] shows a linear response over the entire 0.3- to 1000-nM range with potential linearity above and below this range. The Li-Cor^®^ Pearl Impulse [Fig. 6(b)] shows a linear response over the ∼1.0- to 1000-nM range with no saturation. The PerkinElmer^®^ Solaris [Fig. 6(c)] shows linearity in the ∼2- to 1000-nM range with no saturation. Finally, the Stryker^®^ Spy Elite [Fig. 6(d)] exhibited linearity in the ∼3- to 1000-nM range with saturation starting above ∼1000  nM.

### 3.2 Tissue-Equivalent Depth Sensitivity Testing Standard

**Fig. 7:**
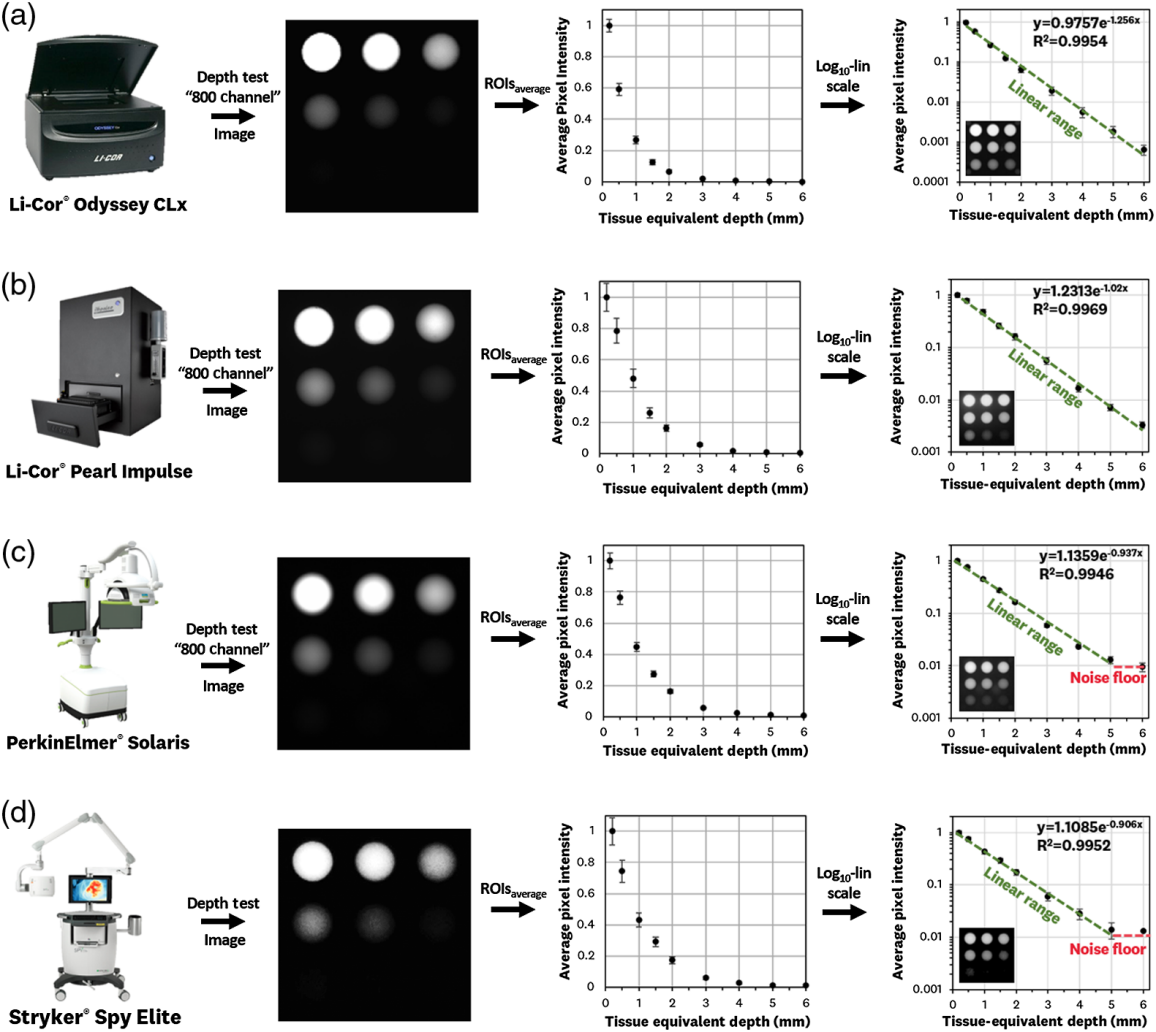
System imaging results for the tissue-equivalent depth test. The test is imaged for the ICG-specific channel of each system and average pixel values of the ROIs corresponding to each well are calculated, normalized, and plotted; the log10–linear scaling plot of these average pixel values allows for the approximation of the depth-sensitivity curve over the 0.2- to 6-mm depth range for (a) Li-Cor^®^ Odyssey CLx “800 channel,” (b) Li-cor^®^ Pearl Impulse “800 channel,” (c) PerkinElmer^®^ Solaris “800 channel,” and (d) Stryker^®^ Spy Elite. The images on the bottom left corner of the log10–linear graphs are the logarithmic pixel representation of the respective fluorescence image.

**Fig. 8:**
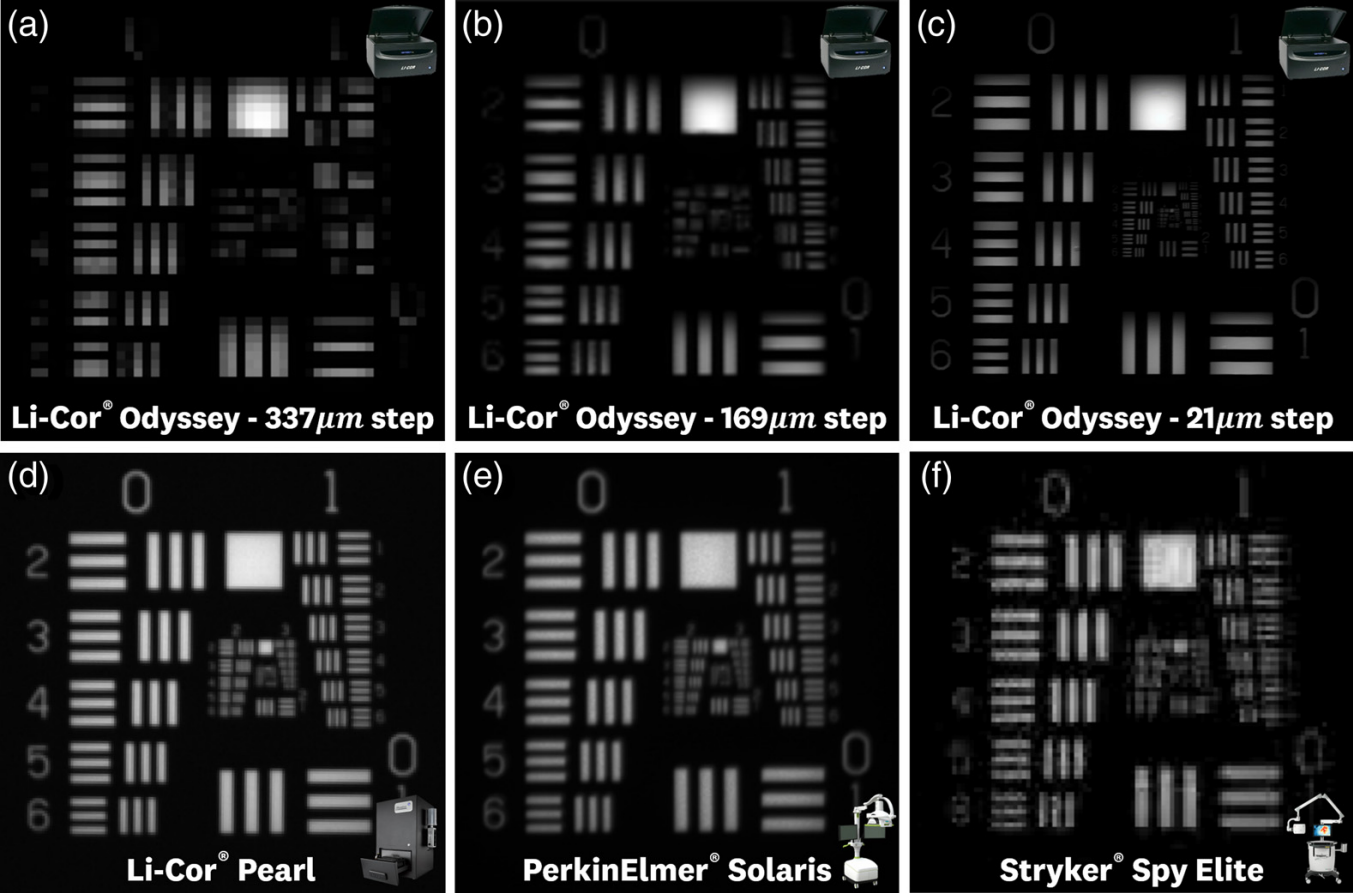
System images of the fluorescence resolution USAF target standard. (a)–(c) Li-Cor^®^ Odyssey “800 channel,” images at varying scanning step sizes of 337, 169, and 21  μm, respectively. (d) Licor-Pearl^®^ “800 channel” image, (e) PerkinElmer^®^ Solaris “800 channel” image, and (f) Stryker^®^ Spy Elite image.

The resulting images and ROI average pixel calculations for the depth sensitivity test are shown in Fig. 7. The average pixel values are normalized to the 0.2-mm depth concentration well for each system. Plotting the average values in the log10–linear plots allows for a visual assessment of a system’s linearity over the 0.2- to 6-mm depth range and an approximation of the depth sensitivity curve. The Li-Cor^®^ Odyssey CLx [Fig. 7(a)] shows linearity over the entire range of depths, with a 90% fall in signal at ∼1.8  mm. The Li-Cor^®^ Pearl Impulse shows linearity over the entire range of depths, with a 90% fall in signal at ∼2.5  mm. The PerkinElmer^®^ Solaris [Fig. 7(c)] shows a linear relationship up to depths of ∼5  mm, with a 90% fall in signal at ∼2.6  mm. The Stryker^®^ Spy Elite [Fig. 8(d)] shows a linear relationship up to depths of ∼5  mm with a 90% fall in signal at ∼2.7  mm.

### 3.3 Fluorescence Resolution Testing Standard

Results from imaging the fluorescence resolution test are shown in Fig. 8. The Li-Cor^®^ Odyssey CLx, given its variable scanning resolution, was set to image at three different step sizes of 337, 169, and 22  μm. The 337-μm step size image resulted in a measured resolution of 354  μm [Fig. 8(a)], 169-μm step size with measured resolution of 177  μm [Fig. 8(b)], and 22-μm step size with measured resolution of 24 and 44  μm for vertical and horizontal directions, respectively, where the horizontal resolution is deteriorated due to motion artifacts of the raster scanning. The Li-Cor^®^ Pearl Impulse image [Fig. 8(d)] resulted in a measured resolution of 99  μm. The PerkinElmer^®^ Solaris image [Fig. 8(e)] resulted in a measured resolution of 157  μm. The Stryker^®^ Spy Elite image resulted in a measured resolution of 223  μm, showing JPEG-like compression artifacts that contribute to a slight degradation in system resolution.

### 3.4 Manufacturing Reproducibility

**Fig. 9:**
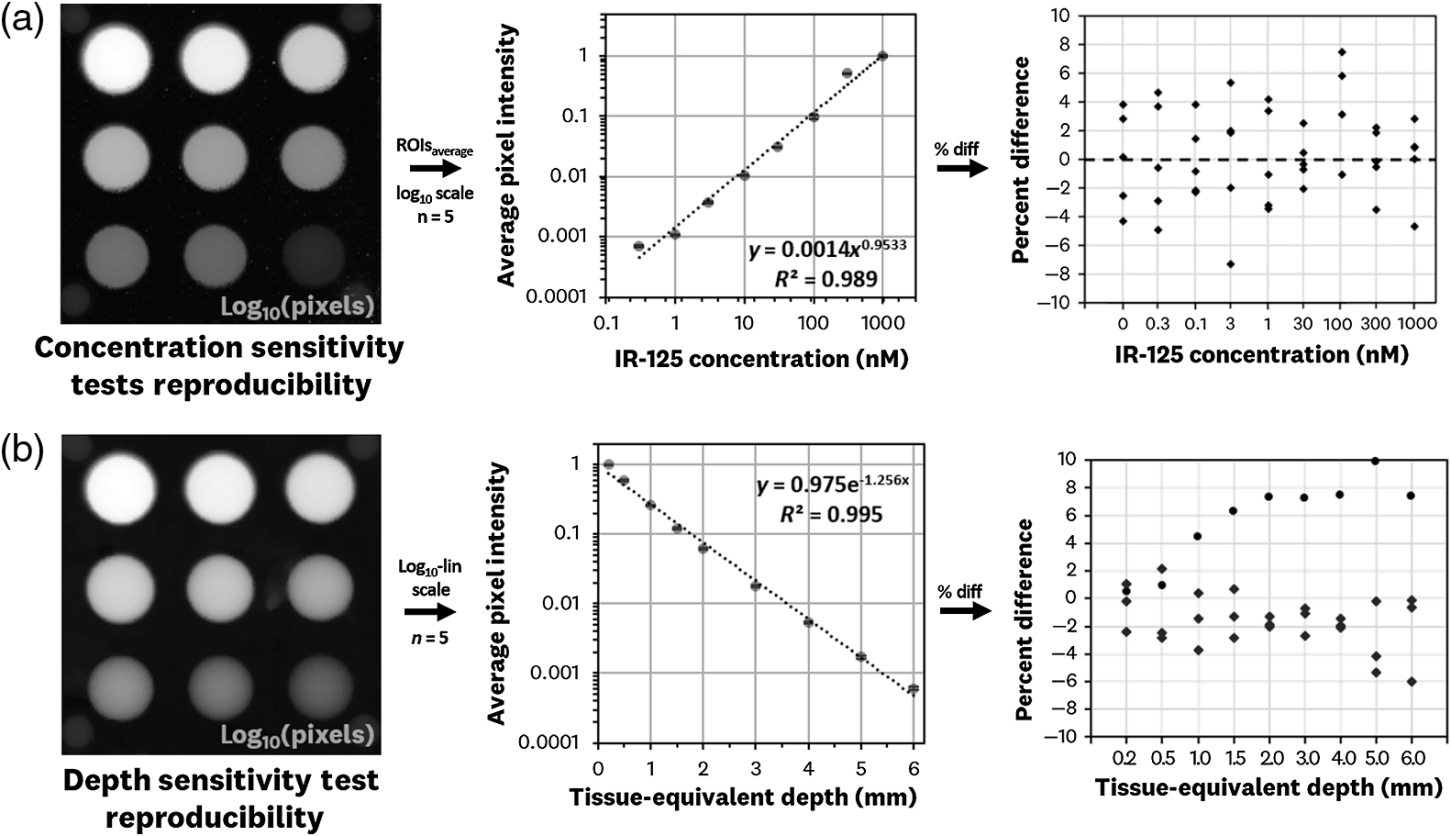
Reproducibility measurements with five manufactured sets of the concentration and depth sensitivity tests using the 800-nm channel of the Li-Cor^®^ Odyssey CLx imaging system: (a) concentration sensitivity test standards were imaged and the average pixel values of each well across the five samples are displayed in a log10–log10 scale, showing a linear relationship with $R^{2}=0.989$ over the 0.3- to 1000-nM range; plotting the percent difference of the five samples shows a random distribution of errors. (b) Depth sensitivity test standards were imaged and the resulting average pixel values of the five samples is displayed in a log10–linear scale showing a linear relationship with $R^{2}=0.995$. Plotting the percent difference of these depth-test samples shows errors in which the deviation sometimes correlates to an individual test standard (top distribution in the percent difference graph).

A total of five sets of the concentration sensitivity and depth sensitivity test were manufactured and measured for insight into the fabrication reproducibility; the results are shown in Fig. 9. The average ROI pixel data for the concentration sensitivity tests is plotted in a log10–log10 scale, showing a linear relationship over the five samples with an $R^{2}=0.989$ over the 0.3- to 3000-nM range [Fig. 9(a)]. The percent difference errors of the concentration tests are randomly distributed. The average ROI data for the depth sensitivity tests are plotted in a log10–linear scale, showing a linear relationship over the five samples with an $R^{2}=0.995$ over the 0.2- to 6-mm depth range [Fig. 9(b)]. The percent difference errors of the depth tests are generally random with a noticeable deviation of values on the higher percentages due to a single sample. This indicates a potential variation within the depth equivalent material of a singular depth test.

## 4 Discussion

### 4.1 Phantom Performance Assessment

Imaging of the ICG-matching standard with all four commercially available systems (Figs. 6–8) showed the ability to benchmark system performance and allow for cross-system comparisons. The concentration sensitivity test allowed for comparison of ICG system detection performance, the tissue-equivalent depth test allowed for comparison of depth-signal curves, and the fluorescence resolution allowed for confirmation of expected resolution values given by device imaging step size and pixel pitch. The results of each test composing the ICG-matching standard are discussed in detail below.

#### 4.1.1 Concentration sensitivity test

The concentration sensitivity test was successfully imaged by all four devices (Fig. 6), showcasing differences in the lowest detectable concentrations between systems. The Li-Cor^®^ Odyssey CLx system was the only system able to achieve linearity across the entire 0.3- to 1000-nM range. Given the use of a control well with 0-nM concentration, we can estimate the expected lowest detectable level of the Odyssey to be ∼0.03  nM through extrapolation of the linear fit. A measured sensitivity of the Odyssey down to 0.1 nM was confirmed with an early prototype of the ICG phantoms. The Stryker^®^ Spy Elite was the only device that showed saturation at the 1000-nM level, where pixel values were maximized in its 8-bit output. The wide range of concentrations used in this test allows for extrapolation of theoretical saturation values of the other three systems through knowledge of each system’s bit depth. Generally, increasing sensitivity scaled with image acquisition time, where the Li-Cor^®^ Odyssey CLx image acquisition time was in the order of minutes while that of the Stryker^®^ Spy Elite was on the order of tenths of seconds. The clinical system (Stryker^®^ Spy Elite) showed the smallest linearity range, which appears to be the result of optimizing sensitivity to concentrations found during intraoperative procedures while providing the video-rate refresh rates needed.

Overall, the ICG-matching concentrations test provided a good balance of probing the noise-floor and saturation values of commercially available systems, with the ability to linearly extrapolate values to determine maximum and minimum detectable concentrations. Furthermore, the concentration sensitivity test allows for cross-system pixel value comparison for correlating ICG concentration readings between different imaging devices.

#### 4.1.2 Tissue-equivalent depth sensitivity test

The tissue-equivalent depth sensitivity test was successfully imaged by all four devices (Fig. 7), showing the expected exponential decay of signal with increased depth. Plotting of the data in a log10–linear scale allows for a visual assessment of a system’s depth-sensitivity curve. The Li-Cor^®^ Odyssey CLx system had the fastest signal decay (90% at ∼1.8  mm), which is most likely attributed to its point-raster imaging modality. The three wide-field imaging systems showed similar depth sensitivity characteristics (90% signal decay at 2.5 to 2.7 mm), which is expected given their use of similar excitation wavelengths and imaging geometries. The depth test allows for characterization of a system’s signal-to-depth decay, which can be used for estimating fluorescence location and, when used alongside the concentration sensitivity test, estimating ICG concentration at a given depth.

#### 4.1.3 Fluorescence resolution test

Imaging of the fluorescence resolution test by the four devices (Fig. 9) confirmed the measured resolutions close to the values provided by the manufacturers (Fig. 5). This test allowed for identification of potential resolution artifacts, including the reduction of horizontal resolution on the Li-Cor^®^ Odessey CLx of ∼20  μm due to raster motion and JPEG-like compression artifacts observed on the Stryker^®^ Spy Elite images. Furthermore, this test can be used to observe potential deterioration of the optical components of a system through resolution changes. Although a simplified method of measuring resolution through determining the smallest line pairs that satisfy the Rayleigh criterion was used, a full contrast transfer function analysis can also be performed for a more detailed look at a system’s optical performance.^19^^,^^35^

### 4.2 Manufacturing Reproducibility

Manufacturing percent error differences between five sets of ICG-matching standards showed errors of <10% for each measurement well. Given that this variation is much less than the percent change for depth and concentration from subsequent wells, a calibration file for each ICG-matching standard can be recorded to accommodate for variations in manufacturability. Using these calibrations would enable cross-system comparison through the correlation of values measured from different ICG-matching standard sets. The next iteration of the standard is currently being designed to be fully 3D printed and manufactured in-house to help reduce percent error differences and manufacturing complexities associated with the polyurethane-based fluorescent mixture.

### 4.3 Improvements to the ICG-Equivalent Standard and Future Directions

The current implementation of the proposed ICG-matching fluorescence imaging standard uses a hybrid manufacturing method of 3D printed molds that incorporate tissue-equivalent material and a polyurethane mixture that incorporates the fluorescent agent. The hybrid manufacturing method outlined here can be used to create spectral-matching phantoms for other fluorescing agents with the ability of designing phantoms that mimic anatomical structures. Development is underway for a fully 3D printed design and manufacturing method that will help reduce variability and complexity in manufacturing. Potential modifications to the standard design include the addition of different depths and concentrations to accommodate for application-specific imaging needs. Long-term stability testing of the standard is underway, where further characterization is needed to accommodate for relative drifts in fluorescence output [currently expected on the range of 1% to 10% over a year (Fig. S1 in the Supplementary Material)]. Implementation of a quantum-dot well is being assessed as a potential solution for calibrating fluorescence intensity decay in the ICG-equivalent wells. Work is also needed in streamlining the calibration of each standard to account for manufacturing variabilities and enable one-to-one cross-system comparison. Further spectral characterization and quantum yield measurements of the ICG-matching concentrations are needed to ensure one-to-one equivalence to in-human ICG concentrations. The current implementation of the ICG-matching phantoms shows promise in becoming a widely adopted fluorescence imaging standard, where addressing the questions posed above and working with both research and industry institutions is necessary to converge on a final design.

## 5 Conclusions

The first long-term stable fluorescence imaging standard that mimics ICG spectral behavior was presented. The ICG-matching standard, composed of a concentration sensitivity, tissue-equivalent depth sensitivity, and fluorescence resolution test, was able to assess the full dynamic sensitivity of four commercially available imaging systems. The outlined manufacturing process, which integrates 3D printed tissue-equivalent material with fluorescence-matching photostable dyes, can be used in applications beyond ICG and NIR imaging. The developed ICG-matching phantom is being tested by research institutions and commercial entities to help assess the overall design, with the hope of advancing toward a widely adopted standard for performance benchmarking and cross-system comparisons.
