# Supplementary material for: Indocyanine green matching phantom for fluorescence-guided surgery imaging system characterization and performance assessment
Source: J Biomed Opt. 2020 May 21;25(5):056003. doi: 10.1117/1.JBO.25.5.056003 (PMC7240319; doi:10.1117/1.JBO.25.5.056003)
Supplement: Supplementary file 1 [file JBO_025_056003_SD001.pdf]

Supplementary Material

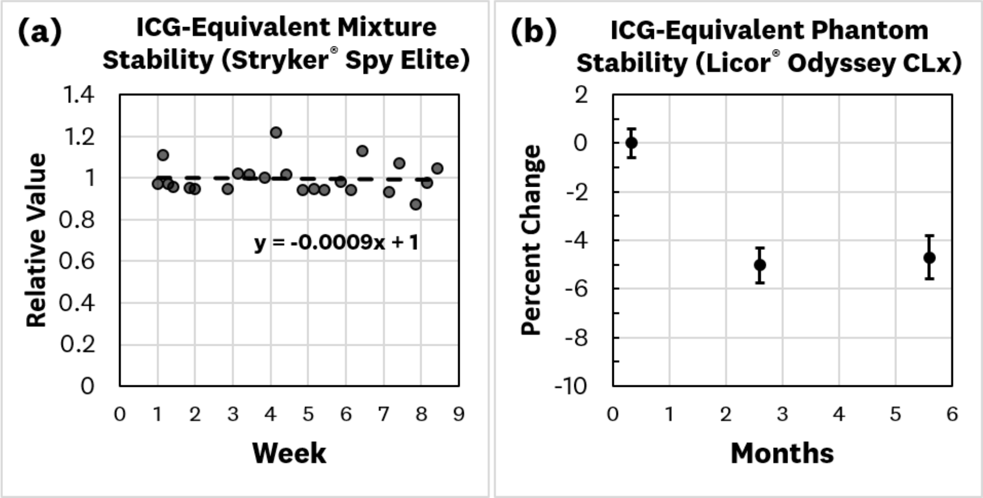

Fig S1. Fluorescence stability measurements of IR-125. (a) Daily measurements of a 100nM IR-125 polyurethane solution using the Stryker Spy Elite over a two month period showing a linear decrease in the measured intensity of 0.09%/week. (b) Percent change measurement of a manufactured concentration phantom using the Licor Odyssey CLx showing a <5% change over a 5 month period. Percent change is averaged over the seven highest concentrations to ensure a high signal to noise.
